# Visualization and bibliometric analysis of 3D printing in cartilage regeneration

**DOI:** 10.3389/fbioe.2023.1214715

**Published:** 2023-06-30

**Authors:** Zhen Yang, Jianwei Li, Haoyuan Deng, Hao Li, Tianyuan Zhao, Tianze Gao, Dan Xing, Jianhao Lin

**Affiliations:** ^1^ Arthritis Clinical and Research Center, Peking University People’s Hospital, Beijing, China; ^2^ Arthritis Institute, Peking University, Beijing, China; ^3^ School of Medicine, Nankai University, Tianjin, China; ^4^ Key Laboratory of Musculoskeletal Trauma and War Injuries PLA, Beijing Key Lab of Regenerative Medicine in Orthopedics, Chinese PLA General Hospital, The First Medical Center, Institute of Orthopedics, Beijing, China

**Keywords:** 3D printing, cartilage regeneration, cartilage repair, tissue engineering, visualization research

## Abstract

The self-repair ability of cartilage defects is limited, and 3D printing technology provides hope for the repair and regeneration of cartilage defects. Although 3D printing technology and cartilage repair and regeneration have been studied for decades, there are still few articles specifically describing the relationship between 3D printing and cartilage defect repair and regeneration, and a bibliometric analysis has not been completed. To supplement, sort out and summarize the content in related fields, we analyzed the research status of 3D printing technology and cartilage repair and regeneration from 2002 to 2022. According to the set search strategy, the Web of Science Core Collection was used as the data source, and the literature search was completed on December 6, 2022. CiteSpace V and VOSviewer were used as bibliometric tools to complete the analysis of the research focus and direction of the published literature. Based on the analysis results, we focus on the occurrence and development of this field of combined medical and engineering research. Moreover, the current advantages and limitations of this field as well as future development prospects are discussed in depth. It will help to shape researchers’ understanding of 3D printing and cartilage repair and regeneration, inspire researchers’ research ideas, guide research directions, and promote related research results to clinical application.

## 1 Introduction

Cartilage is a layer of connective tissue with good elasticity covering the surface of joints, which plays a role in bearing composite, providing lubrication, abrasion resistance and buffering in joint movement (Huber et al., 2000). Cartilage is mainly composed of chondrocytes and a dense extracellular matrix. Due to the lack of nerves and blood vessels in cartilage, cartilage loses the ability to heal itself, so defects are irreversible injuries (McGonagle et al., 2017). The persistence of cartilage defects will lead to the impairment of joint mobility, manifested as joint stiffness and pain, and eventually further develop into osteoarthritis (Kwon et al., 2019). Osteoarthritis is an irreversible degenerative disease, and the ultimate treatment is joint replacement. It is a major health problem and has a serious impact on the physical and mental health of patients (Latourte et al., 2020). How to repair or regenerate cartilage defects and restore the original biological function of injured cartilage is still a serious clinical challenge.

Currently, no treatment is available to repair damaged cartilage. The main effect of symptomatic treatment with anti-inflammatory drugs or analgesics commonly used in the clinic is to reduce pain (Matsiko et al., 2013). Common surgical methods for the treatment of cartilage defects include microfracture surgery, autologous osteochondral transplantation, allogeneic osteochondral transplantation, autologous chondrocyte implantation (ACI), matrix-induced autologous chondrocyte implantation (MACI) and joint replacement (Krych et al., 2020). There are still many limitations in these clinical treatments, such as fibrocartilage production and immune rejection, which cannot fully meet clinical needs.

Tissue engineering is a very promising strategy to repair cartilage defects with the development of scaffolds that mimic native tissue as a framework for cell adhesion and proliferation to replace the site of injury (Makris et al., 2015). 3D printing technology is based on computer-aided and three-dimensional molding methods, which allow the precise assembly of biomaterials and cells and the construction of designed 3D models by deposition as needed to achieve personalized structure and function (Vijayavenkataraman et al., 2018). Natural cartilage tissue has a complex multilayered structure, and there is no obvious boundary between the layers. The cell morphology and extracellular matrix composition of the layers are different (Huber et al., 2000; Zhang et al., 2020). Complex biomimetic structures are difficult to reconstruct by conventional engineering methods, while 3D printing technology has great potential in preparing complex 3D scaffolds with similar structures to natural tissue, which is well suited for cartilage tissue engineering (Cui et al., 2017). 3D printing technology can achieve functional regeneration of tissues by finely imitating the physiological structure and even biological function of natural tissues (Mouser et al., 2017). 3D printing technology not only has the characteristics of high precision but can also be used to control the size, shape, and aperture arrangement of the scaffold according to the requirements of computer software (Fazal et al., 2021). In addition, different materials or bioinks with different contents also provide more solutions for the controllable personalization of 3D printed scaffolds (Singh et al., 2019). 3D printed products can be used as excellent scaffold materials in tissue engineering strategies, providing a suitable microenvironment for subsequent seed cell regeneration while allowing the incorporation of growth factors for biological regulation (Yang et al., 2022). We believe that with the deepening of physiological research and the continuous development of 3D printing technology, tissue engineering technology for clinical use in the future will be refined and personalized. 3D printing technology has a unique charm in the field of cartilage repair and regeneration, which attracts researchers to explore continuously.

In the past two decades, 3D printing technology has made great progress, but research on 3D printing technology to repair cartilage defects has not been systematically summarized. The characteristics of bibliometric analysis are that it can qualitatively and quantitatively analyze the influence of journals, institutions, research teams, researchers or countries on the research field and then describe the research status and predict the development trend of related fields (Krauskopf, 2018). Therefore, we used bibliometric analysis to analyze the relevant literature, and then discussed in depth the current advantages and limitations of the field as well as the future development prospects, hoping that its contents will have a positive impact on the development of 3D printing for cartilage defect repair and provide ideas for relevant researchers.

## 2 Materials and methods

We completed a literature search on 6 December 2022, using the Web of Science Core Collection as the data source, and the search terms were as follows: theme = 3D bioprinting OR 3D printing OR 3D printed OR 3D print OR three-dimensional bioprinting OR three-dimensional printing OR three-dimensional printed OR three-dimensional print *AND all fields = cartilage regeneration OR cartilage repair OR cartilage injury OR chondral regeneration OR chondral repair OR chondral injury*AND publishing year = (01 December 2002 to 01 December 2022). We used common bibliometric indicators in the scientific community to evaluate the obtained literature, such as total citations, average citations, and H-index (Hirsch, 2005). We obtained journal impact factors (IF) from Journal Citation Reports 2021 for analysis. We chose VOSviewer (Leiden University, Leiden, Netherlands) software to build and visualize the bibliometric network of publications in our study (van Eck and Waltman, 2010). Different items are represented by different nodes, the size of nodes indicates the number of publications, the color of nodes indicates the publication year, and the thickness of the line between nodes indicates the strength of collaboration or integration. Citespace (6.1. R2) developed by Professor Chen C was used for country/region and institution collaboration analysis, journal double graph superposition analysis, author collaboration and cocited authors analysis, cocited literature and keyword cluster detection, and intensive outbreak citation literature and keyword analysis (Chen, 2016). Specific analysis parameters included link retention factor (LRF = 3), year of review (LBY = 5), e (*N* = 1), time span (2012–2022), year per slice (1), link (strength: cosine, range: slice), selection criteria (g-index: *k* = 25), and minimum duration (key words MD = 2; MD = 5 as a reference).

## 3 Result

### 3.1 Analysis of global literature publication trend

**FIGURE 1:**
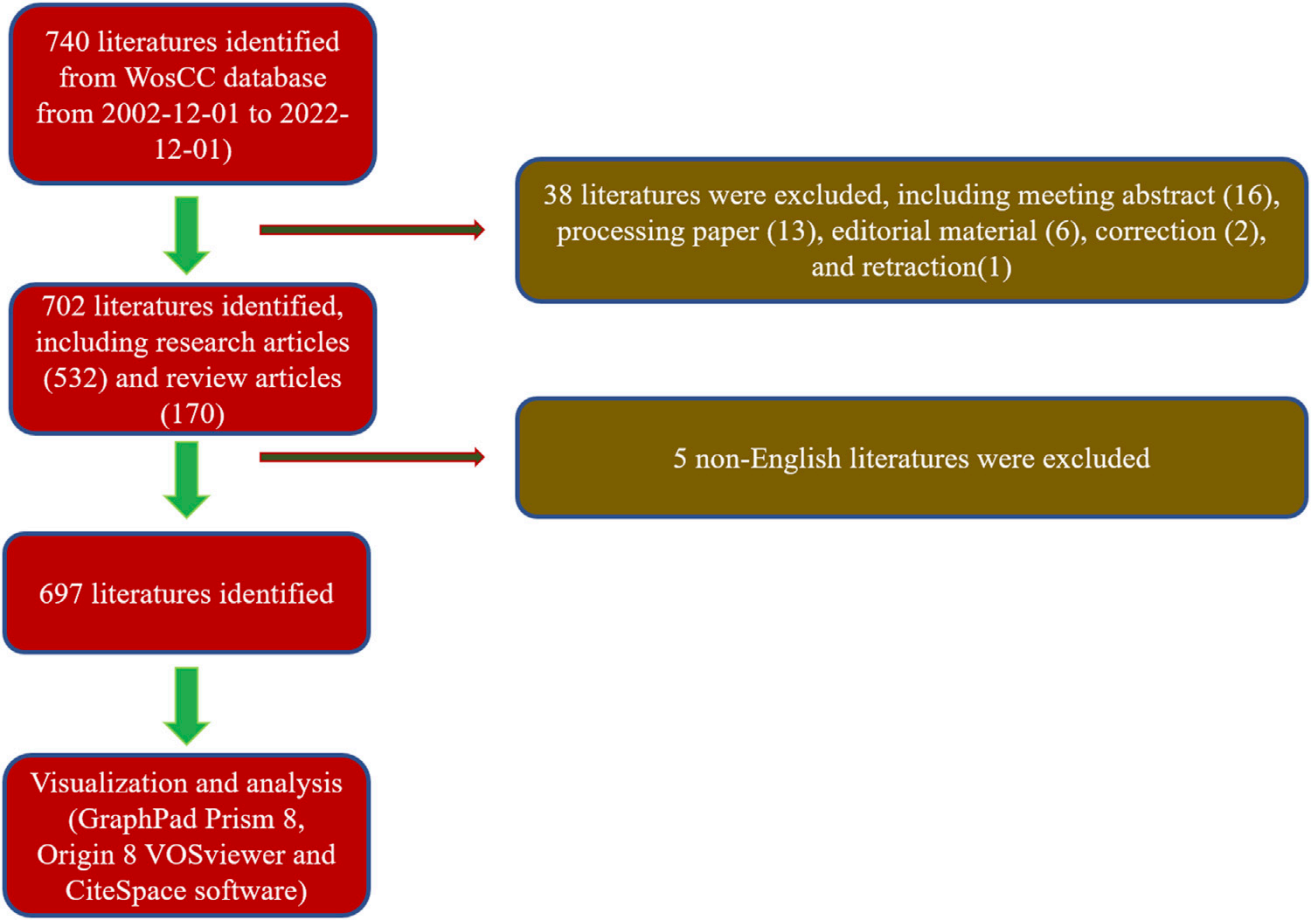
Flowchart depicting the article selection process.

**FIGURE 2:**
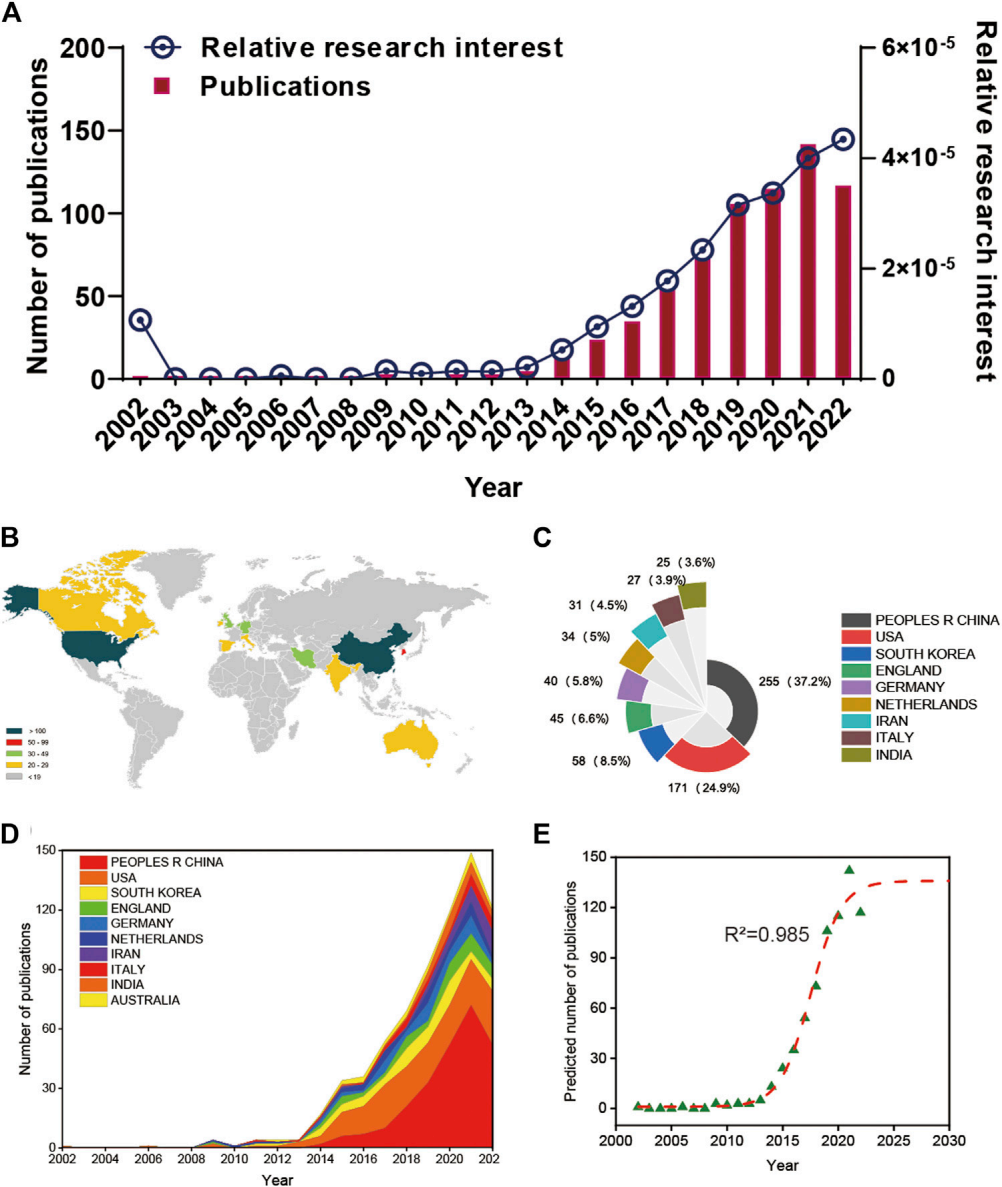
Global publishing trends and countries/regions contributing to 3D printing in cartilage regeneration and repair. **(A)** The annual number of publications related to 3D printing in cartilage regeneration and repair. **(B)** A world map depicting the distribution of 3D printing in cartilage regeneration and repair. **(C)** The sum of 3D printing in cartilage regeneration- and repair-related publications in the top 10 countries/regions. **(D)** The annual number of publications in the top 10 most productive countries from 2002 to 2022. **(E)** Model fitting curves of global trends in publications of 3D printing in cartilage regeneration and repair.

A total of 740 articles were collected from the Web of Science database. Of these, 38 articles were excluded, including meeting abstracts (16), processing papers (13), editorial materials (6), corrections (2), and retractions (1). In addition, 5 non-English studies were excluded. Finally, 697 articles met the inclusion criteria for using the Web of Science database (Figure 1). We summarized the global literature trend (Figure 2). The annual publication number of relevant literature has not exceeded a single place from 2002 to 2013 and has steadily increased from 13 to 73 from 2014 to 2018. Since 2019, the annual publication number of relevant literature has exceeded 100, with the highest being 142 in 2021. This is almost twice the number in 2018. A total of 54 countries/regions around the world have published English literature in this field. The top five countries with the largest number of articles were China (255 articles, 36.585%), United States (171 articles, 24.534%), Republic of Korea (58 articles, 8.321%), United Kingdom (45 articles, 6.456%), and Germany (40 articles, 5.739%). The annual number of publications of the top 10 countries/regions increased from 1 in 2002 to 149 in 2021 and then fell back to 121 in 2022 (Figure 2D). The reason for the decline in the number of publications may be related to the time of statistics, and there may still be some unpublished articles in 2022 that have not been included in the statistics. The fitting curve of the global publication trend (Figure 2E) has a correction coefficient R2 of 0.985, and this time curve created by the logistic regression model can predict the future global publication trend. According to the forecast results, the number of publications is expected to reach 136 by 2025. In general, in recent years, research on 3D printing and cartilage repair and regeneration has received increasing attention from researchers and is developing rapidly.

### 3.2 Citation analysis of global literature

**FIGURE 3:**
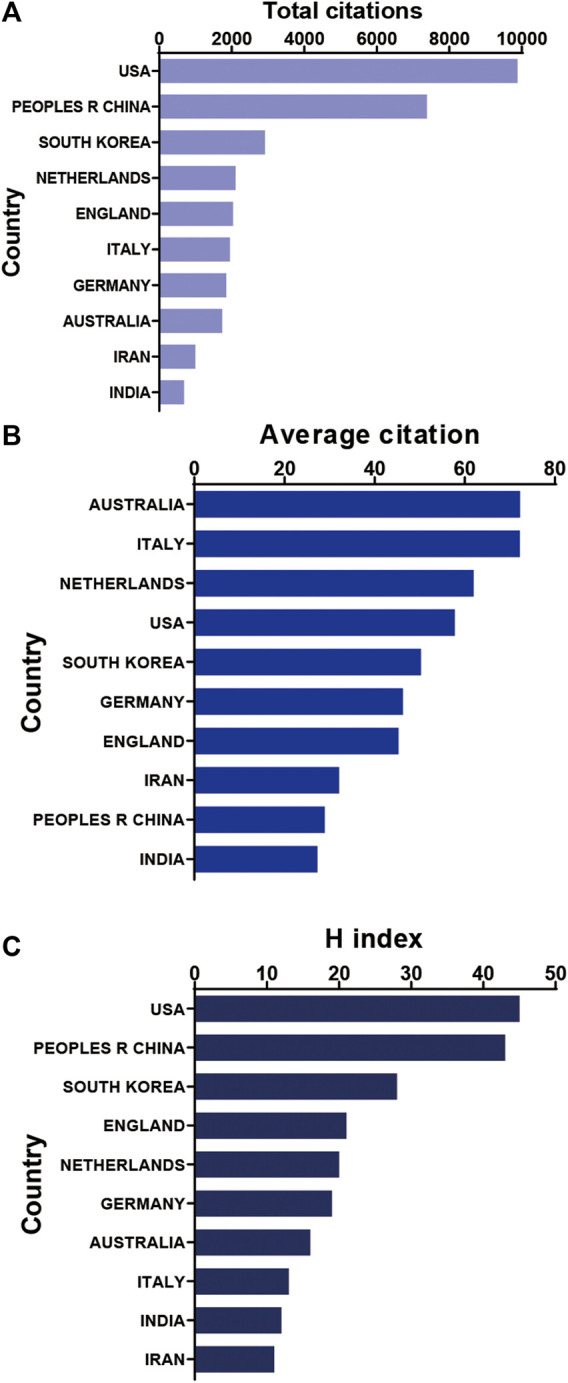
Citation frequency and H-index levels of different countries/regions. **(A)** The top 10 countries and regions of total citations of 3D printing in cartilage regeneration and repair. **(B)** The top 10 countries and regions of the average citations per paper in 3D printing in cartilage regeneration and repair. **(C)** The top 10 countries and regions of the H-index of 3D printing in cartilage regeneration and repair.

It shows the citation frequency of different countries/regions (Figure 3A), in which the United States has the highest citation frequency (9,880), and China ranks behind the United States (7,385), far ahead of Republic of Korea (2,918), Netherlands (2,106) and the United Kingdom (2,038). Among the top 10 countries and regions with the highest average citation frequency (Figure 3B), Australia has the highest average citation frequency (72.25), followed by Italy (72.22), Netherlands (61.94), United States (57.78) and Republic of Korea (50.31). We analyzed the top 10 countries with the highest H-index in the relevant publications (Figure 3C), with the United States (45) and China (43) leading Republic of Korea (28), the United Kingdom (21) and Netherlands (20).

### 3.3 Analysis of national and institutional cooperation in global literature

**FIGURE 4:**
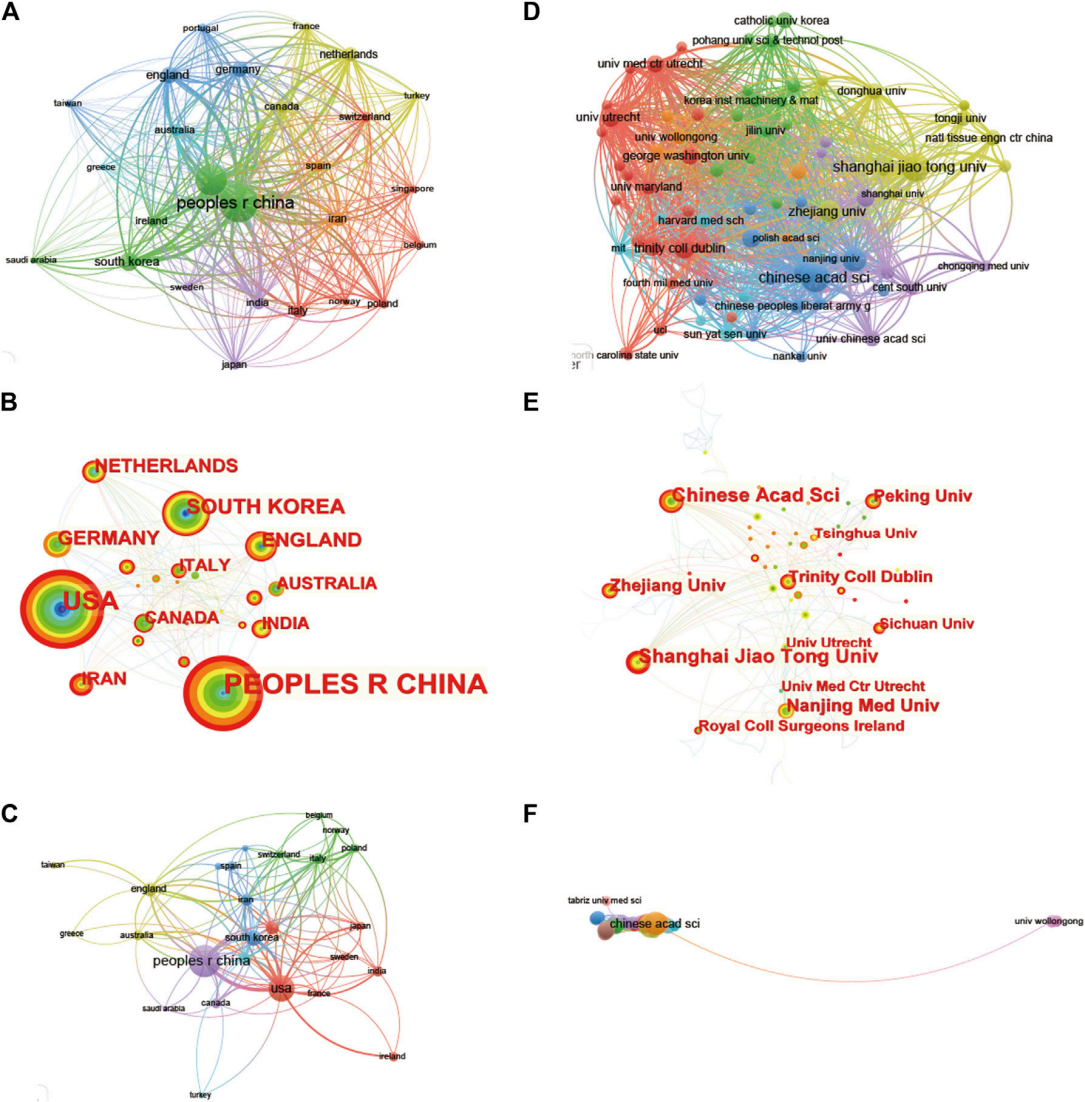
Mapping of countries/regions and institutions associated with 3D printing in cartilage regeneration and repair. Country/regional collaboration analysis based on Vosviewer **(A)** and Citespace **(B)**. **(C)** Mapping of the 26-country coauthorship analysis on 3D printing in cartilage regeneration and repair. Institutional collaboration analysis based on Vosviewer **(D)** and Citespace **(E)**. **(F)** Mapping of the 72-institution coauthorship analysis on 3D printing in cartilage regeneration and repair. The nodes represent countries/regions or institutions, and the lines connect them. The number of publications grows proportionally to the size of the nodes. The lines between the nodes represent the cooperation relationship, and the thickness of the connecting lines represents the strength of their cooperation; the closer the cooperation, the thicker the connecting lines. The nodes with the outermost orange circles have higher centrality. From 2002 to 2022, the color changes from green to orange.

It can be seen in the bibliography coupling map in Figure 4A that China (114,502) and United States (104,244) have similar total link strength, followed by United Kingdom (36,232), Republic of Korea (33,932), and Iran (33,932). China (254) has the highest production, followed by United States (171), Republic of Korea (58), United Kingdom (45) and Germany (40) (Figure 4B). United States, China, United Kingdom and Netherlands have a relatively close cooperative relationship (Figure 4C). Table 1 lists the top 10 institutions that have published the most relevant literature. The first is the Chinese Academy of Sciences, followed by Shanghai Jiao Tong University and Nanjing Medical University. The same results can also be seen in Figures 4D, E. Among them, Shanghai Jiao Tong University, Chinese Academy of Sciences, National Tissue Engineering Research Center of China and Peking Universities are relatively closely connected (Figure 4F).

**TABLE 1:** The top 10 institutions published literature related to 3D printing in cartilage regeneration and repair.

Rank	Institution	Article counts	Percentage (N/740)	Country	Total citations	Average citation	H-index
1	Chinese Academy of Sciences	40	5.739	China	1,679	41.98	22
2	Shanghai Jiao Tong University	37	5.308	China	1,147	28.68	17
3	Nanjing Medical University	26	3.73	China	916	35.23	17
4	Peking University	25	3.587	China	869	33.42	13
5	Zhejiang University	22	3.156	China	659	29.95	12
6	Trinity College Dublin	21	3.013	Ireland	1,099	52.33	13
7	Utrecht University	21	3.013	Netherland	1885	89.76	16
8	Royal College of Surgeons Ireland	20	2.869	Ireland	958	47.9	12
9	Utrecht University Medical Center	20	2.869	Netherland	1865	93.25	15
10	Harvard University	16	2.296	United States	1,124	62.44	8

### 3.4 Analysis of journals and research fields of global literature

**FIGURE 5:**
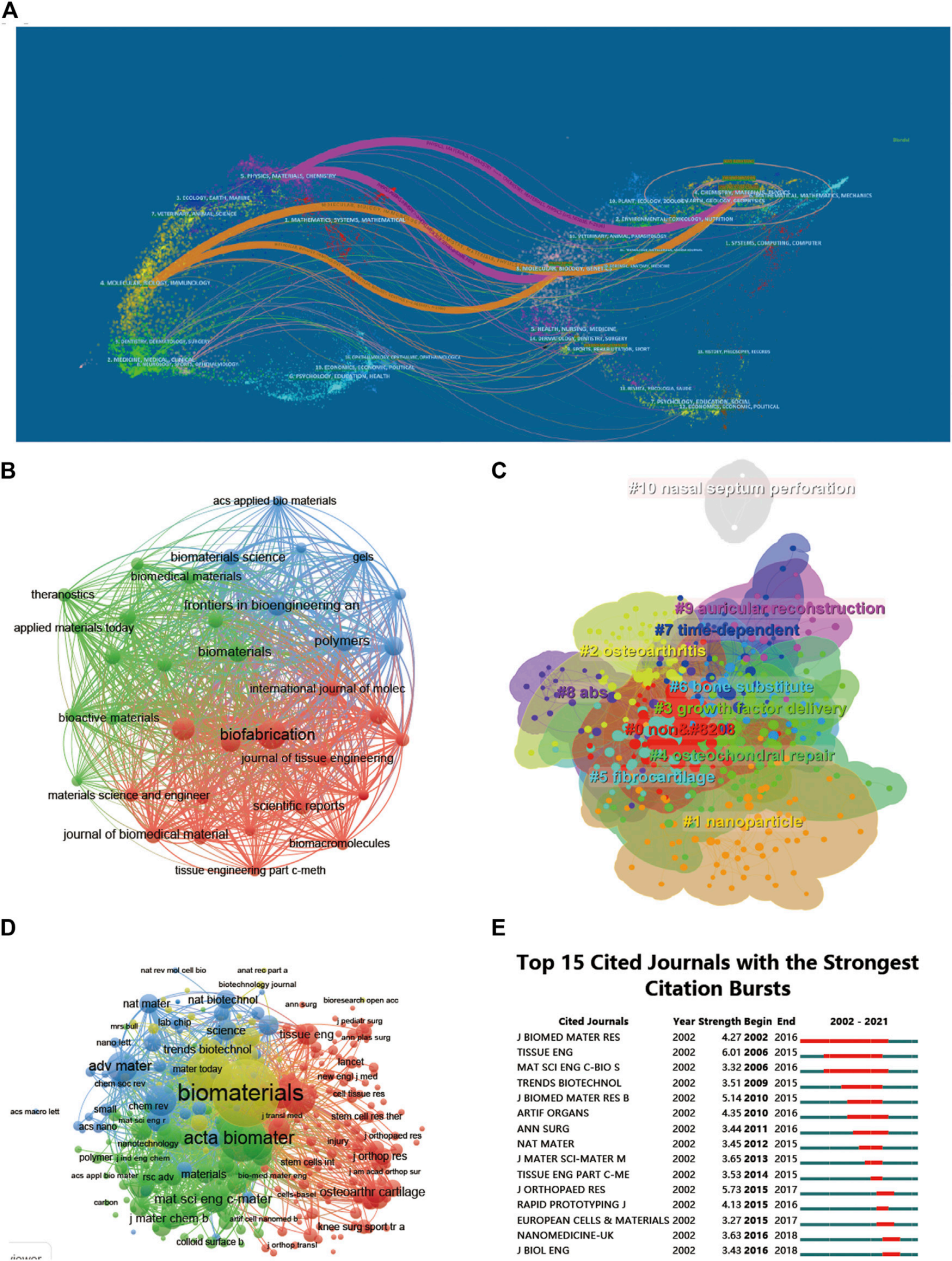
Articles published and cited in different journals on 3D printing in cartilage regeneration and repair. **(A)** The dual-map overlay of journals related to 3D printing in cartilage regeneration and repair. **(B)** Mapping of the identified journals based on Vosviewer. **(C)** Clustering analysis of the cocited journal network based on CiteSpace. **(D)** Mapping of the cocited journals related to this field. **(E)** Top 15 journals with the strongest citation bursts of publications related to 3D printing in cartilage regeneration and repair.

Among the top 10 journals with the most publications included in this study (Table 2), biofabrication (impact factor = 11.061, 2022) has the largest number of publications (40). Much higher than Acta Biomaterialia with 24 publications (impact factor = 10.633, 2022), Frontiers in Bioengineering and Biotechnology (impact factor = 6.064, 2022) came in third with 19 publications, Advanced Healthcare Materials (impact factor = 11.092, 2022), Biomaterials (impact factor = 15.304, 2022) and Polymers (impact factor = 4.967, 2022) each had 18 publications. We have listed ten representative research areas related to 3D printing in cartilage regeneration and repair (Table 3). Among them, Materials Science, Engineering, Science Technology Other Topics and Chemistry ranked in the top four, leading the other fields. In addition, a double map overlay of journals was used to analyze the association between cited journals and subject categories among cited journals (Figure 5A). The left to right spline wave describes the citation path, and this interaction illustrates the connection between different areas of research. The main citation path is represented by two pink and orange paths. We visualized the literature cited by different journals (Figure 5B) and performed coclustering analysis via CiteSpace (Figure 5C). Nanoparticles, osteoarthritis, growth factor delivery, osteochondral repair and fibrocartilage were the research hotspots. In the citation relationship between different journals (Figure 5D), the top five journals with the highest total connection strength among 4,559 journals were Biomaterials (total connection strength = 394,915 times), Biofabrication (total connection strength = 219,751 times), and Biofabrication (total connection strength = 219,751 times). Acta Biomaterialia (total link strength = 215,651), Advanced Healthcare Materials (total link strength = 111,474) and Advanced Materials (total link strength = 106,342). We also listed the top 15 journals with the highest citation rate for publications related to 3D printing in cartilage regeneration and repair (Figure 5E).

**TABLE 2:** The top 10 productive journals related to 3D printing in cartilage regeneration and repair.

Rank	Journal	Article counts	Percentage (N/740)	Citation per article	H-index	IF
1	Biofabrication	40	5.739	57.25	25	11.061
2	Acta Biomaterialia	24	3.443	32.07	13	10.633
3	Frontiers in Bioengineering and Biotechnology	19	2.726	70.13	17	6.064
4	Advanced Healthcare Materials	18	2.582	53.39	14	11.092
5	Biomaterials	18	2.582	134.78	13	15.304
6	Polymers	18	2.582	17.56	8	4.967
7	Materials	17	2.439	32.82	11	3.748
8	Tissue Engineering Part A	15	2.152	32.07	13	4.080
9	Scientific Reports	14	2.009	57	12	4.996
10	Journal of Biomedical Materials Research Part B Applied Biomaterials	13	1.865	25.08	8	3.405

**TABLE 3:** The top 10 well-represented research areas related to 3D printing in cartilage regeneration and repair.

Rank	Research areas	Records	Percentage (N/2025)	Total citations	Citation per article	H-index
1	Materials Science	371	53.228	16,518	42.14	65
2	Engineering	262	37.59	13,012	45.34	58
3	Science Technology Other Topics	124	17.791	5,874	45.53	41
4	Chemistry	105	15.065	4,740	43.09	38
5	Cell Biology	79	11.334	3,336	35.87	28
6	Polymer Science	60	8.608	1,018	16.97	18
7	Physics	57	8.178	3,131	52.18	27
8	Biotechnology Applied Microbiology	50	7.174	2,834	53.47	24
9	Biochemistry Molecular Biology	44	6.313	2,309	52.48	18
10	Research Experimental Medicine	33	4.735	1,287	37.85	19

### 3.5 Author collaboration analysis

**FIGURE 6:**
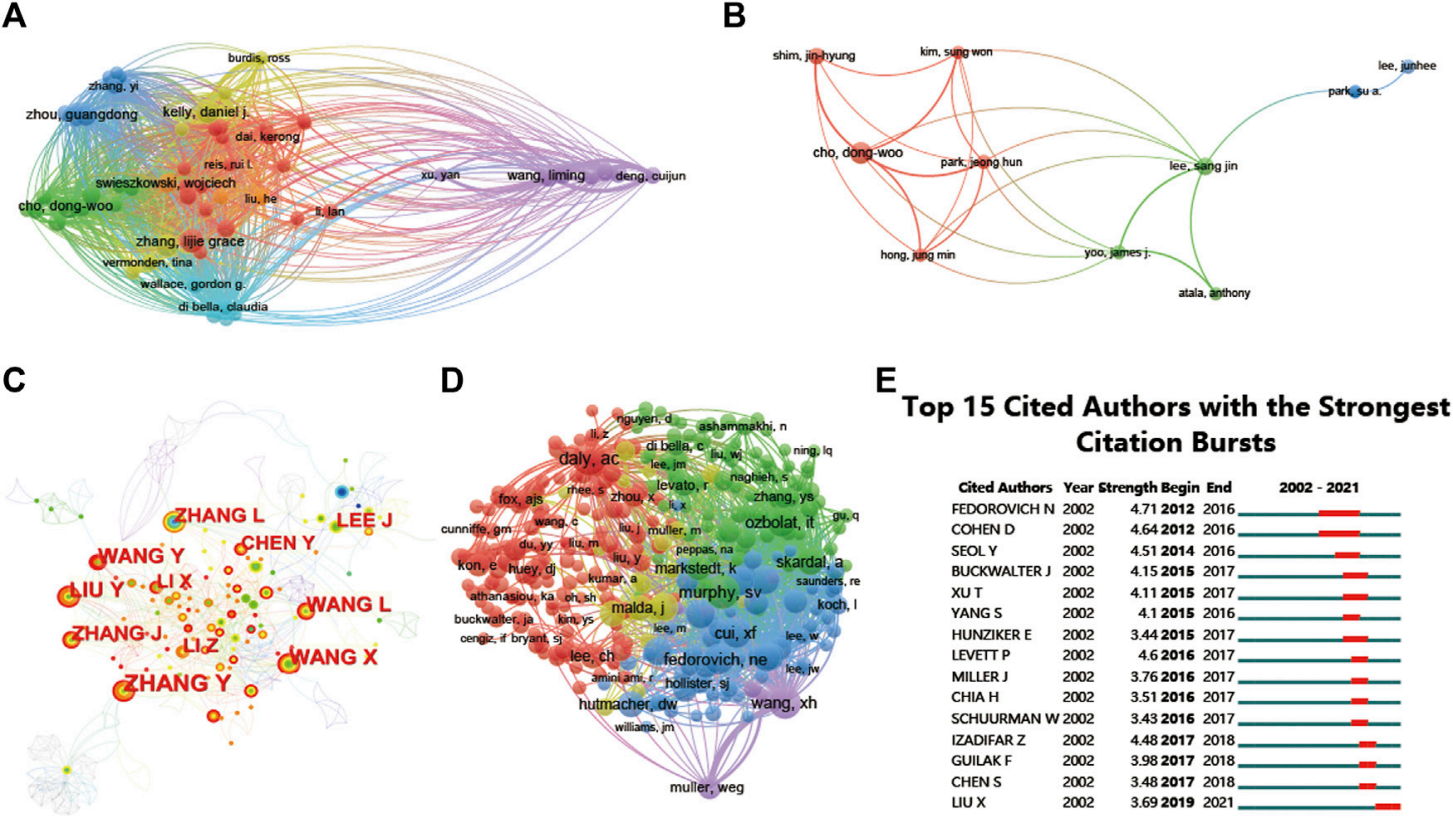
Mapping of authors in studies on 3D printing in cartilage regeneration and repair. **(A)** Mapping of the identified authors in this field. **(B)** Mapping of the 10-author coauthorship analysis in this field. **(C)** Author collaboration analysis based on CiteSpace. **(D)** Network visualization diagram of the cocited authors of the publications. **(E)** Top 15 cited authors with the strongest citation bursts of publications related to 3D printing in cartilage regeneration and repair. Author collaboration or cocited authors are indicated by the node. The cocitation relationship is indicated by the line connecting the nodes. The node area grows as the number of cocitations increases. The colors represent different years. In **(C)**, the color changes from green to orange from 2002 to 2022.

The 697 articles we collected included 3,413 authors. We have visualized the collaborative relationships among authors of literature in this field (Figure 6A), and the cocitation relationships of the top 10 authors are highlighted (Figure 6B). The collaborative relationships among relevant authors are also visualized (Figure 6C). The top five authors with the highest total connection strength were daly, ac, murphy, sv, wang, xh, cui, xf, and fedorovich, and they had the highest total connection strength (Figure 6D). Citation bursts is a valuable indicator that an author is frequently cited in a particular field over a period of time. Top 15 cited authors with the strongest citation bursts of publications related to 3D printing in cartilage regeneration and repair (Figure 6E). In first place is “FEDOROVICH N” (strength = 4.71), followed by “COHEN D.” Moreover, “FEDOROVICH N” and “COHEN D” both had the longest outbreak time (2012–2016). The top 10 authors with the most publications and citations on 3D printing in cartilage regeneration and repair (Table 4). We similarly summarized the top 10 sources of funding to support authors in research related to 3D printing in cartilage regeneration and repair (Table 5).

**TABLE 4:** The top 10 authors with the most publications and citations on 3D printing in cartilage regeneration and repair.

Rank	Highly published authors	Article counts	Article counts (N/740)	Country	Total citations
1	Kelly DJ	18	2.582	England	1,084
2	Malda J	17	2.439	Netherland	1,674
3	Lee SJ	15	2.152	United States	625
4	Cho DW	14	2.009	Republic of Korea	1,355
5	Zhou GD	13	1.865	China	372
6	Wang LM	11	1.578	China	516
7	Xu Y	11	1.578	China	447
8	Yao QQ	11	1.578	China	572
9	Zhang LG	11	1.578	China	701
10	Zhang Y	11	1.578	China	1,514

**TABLE 5:** The top 10 funding sources related to 3D printing in cartilage regeneration and repair.

Rank	Funds	Records	Percentage (N/740)	Country
1	National Natural Science Foundation of China Nsfc	169	24.247	China
2	United States Department of Health Human Services	63	9.039	United States
3	National Institutes of Health Nih Usa	62	8.895	United States
4	National Key Research and Development Program of China	42	6.026	China
5	European Commission	39	5.595	European
6	National Key R D Program of China	30	4.304	China
7	National Science Foundation Nsf	28	4.017	China
8	European Research Council Erc	22	3.156	European
9	Nih National Institute of Biomedical Imaging Bioengineering Nibib	22	3.156	United States
10	Fundamental Research Funds for the Central Universities	21	3.013	China

### 3.6 Reference analysis

**FIGURE 7:**
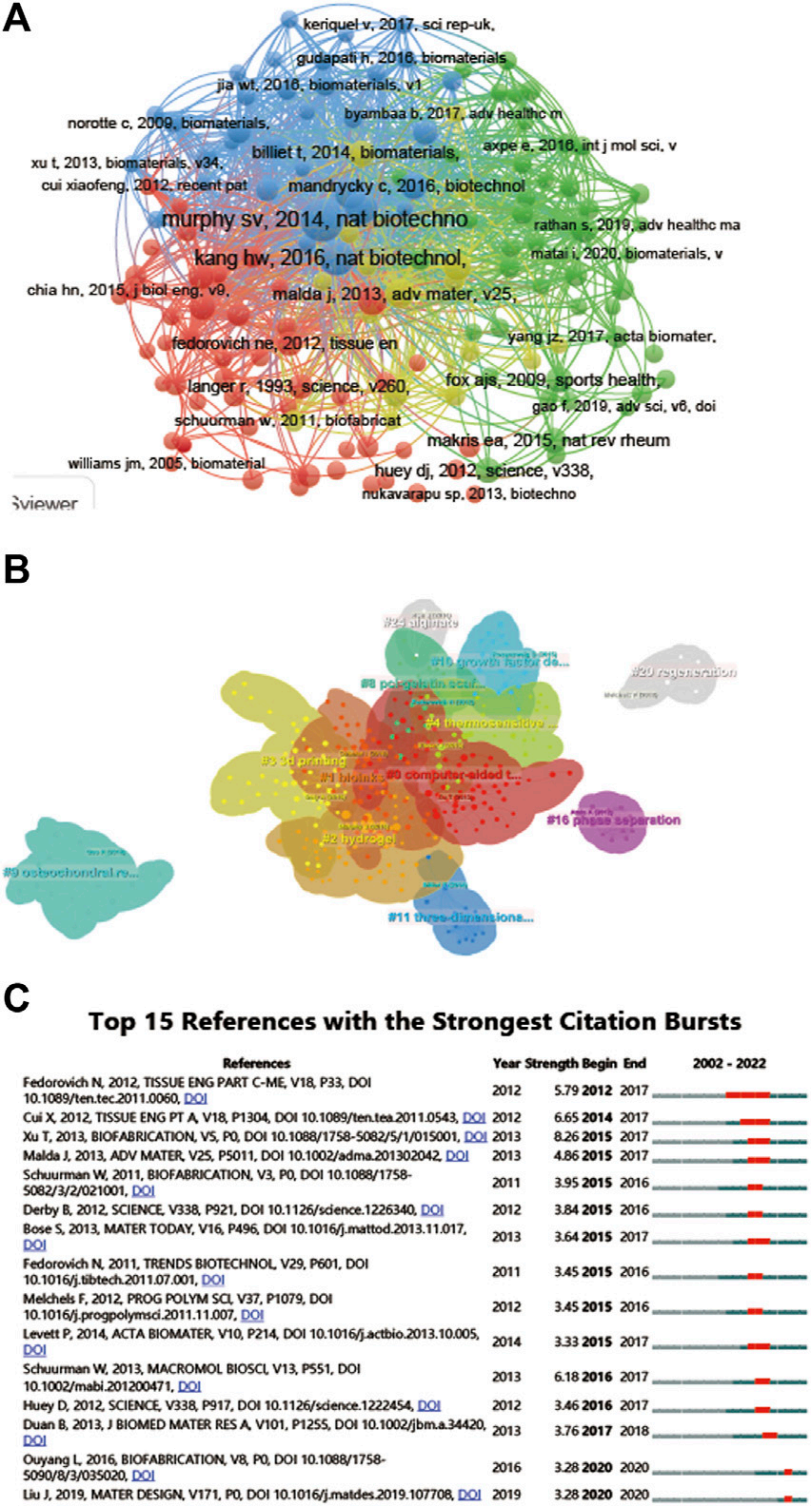
Mapping of cited references in studies on 3D printing in cartilage regeneration and repair. **(A)** Mapping of the cocited references related to this field. **(B)** Clustering analysis of the cocited reference network based on CiteSpace. **(C)** Top 15 references with the strongest citation bursts of publications related to 3D printing in cartilage regeneration and repair.

A total of 144 of the 31,422 citations were cited more than 20 times (Figure 7A). Among the top 5 most cited review articles (Table 6), “Recent advances in 3D printing of biomaterials” was cited 965 times, followed by “Scaffolds for Bone Tissue Engineering: State of the art and new perspectives,” which was cited 608 times, and “Bone regenerative medicine: classic options, novel strategies, and future directions” was cited 596 times. Among the top 5 most cited research articles (Table 7), “Scaffold free vascular tissue engineering using bioprinting” was cited 853 times, “A three-dimensional osteochondral composite scaffold for articular cartilage repair” was cited 496 times, and “Reinforcement of hydrogels using three-dimendimensionally printed microfibers” was cited 454 times. We conducted a visual analysis of the keywords in the references (Figure 7B) and found that computer, bioinks, hydrogel are hot spots in the references. In our study, CiteSpace identified the top 25 references with the most citation bursts (Figure 7C).

**TABLE 6:** The top 5 review articles with the most citations in the field of 3D printing in cartilage regeneration and repair.

Rank	Title	Corresponding author	Journal	IF	Publication year	Total citations
1	Recent advances in 3D printing of biomaterials	Wu, BM	Journal of Biological Engineering	6.248	2015	965
2	Scaffolds for Bone Tissue Engineering: State of the art and new perspectives	Grigolo, B	Materials Science and Engineering C-Materials for Biological Applications	8.457	2017	608
3	Bone regenerative medicine: classic options, novel strategies, and future directions	Maffulli, N	Journal of Orthopedic Surgery and Research	2.677	204	596
4	Tissue Engineering and Regenerative Medicine: History, Progress, and Challenges	Yarmush, ML	Annual Review of Chemical and Biomolecular Engineering	9.7	2011	358
5	Cell-laden hydrogels for osteochondral and cartilage tissue engineering	Khademhosseini, A	Acta Biomaterialia	10.633	2017	335

**TABLE 7:** The top 5 research articles with the most citations in the field of 3D printing in cartilage regeneration and repair.

Rank	Title	Corresponding author	Journal	IF	Publication year	Total citations
1	Scaffold-free vascular tissue engineering using bioprinting	Forgacs, G	Biomaterials	15.304	2009	853
2	A three-dimensional osteochondral composite scaffold for articular cartilage repair	Ratcliffe, A	Biomaterials	15.304	2002	469
3	Reinforcement of hydrogels using three-dimensionally printed microfibers	Malda, J	Nature Communications	17.694	2015	454
4	Direct Human Cartilage Repair Using Three-Dimensional Bioprinting Technology	D'Lima, DD	Tissue Engineering Part A	4.08	2012	404
5	An additive manufacturing-based PCL-alginate-chondrocyte bioprinted scaffold for cartilage tissue engineering	Cho, DW	Journal of Tissue Engineering And Regenerative Medicine	4.323	2015	308

### 3.7 Keyword analysis

**FIGURE 8:**
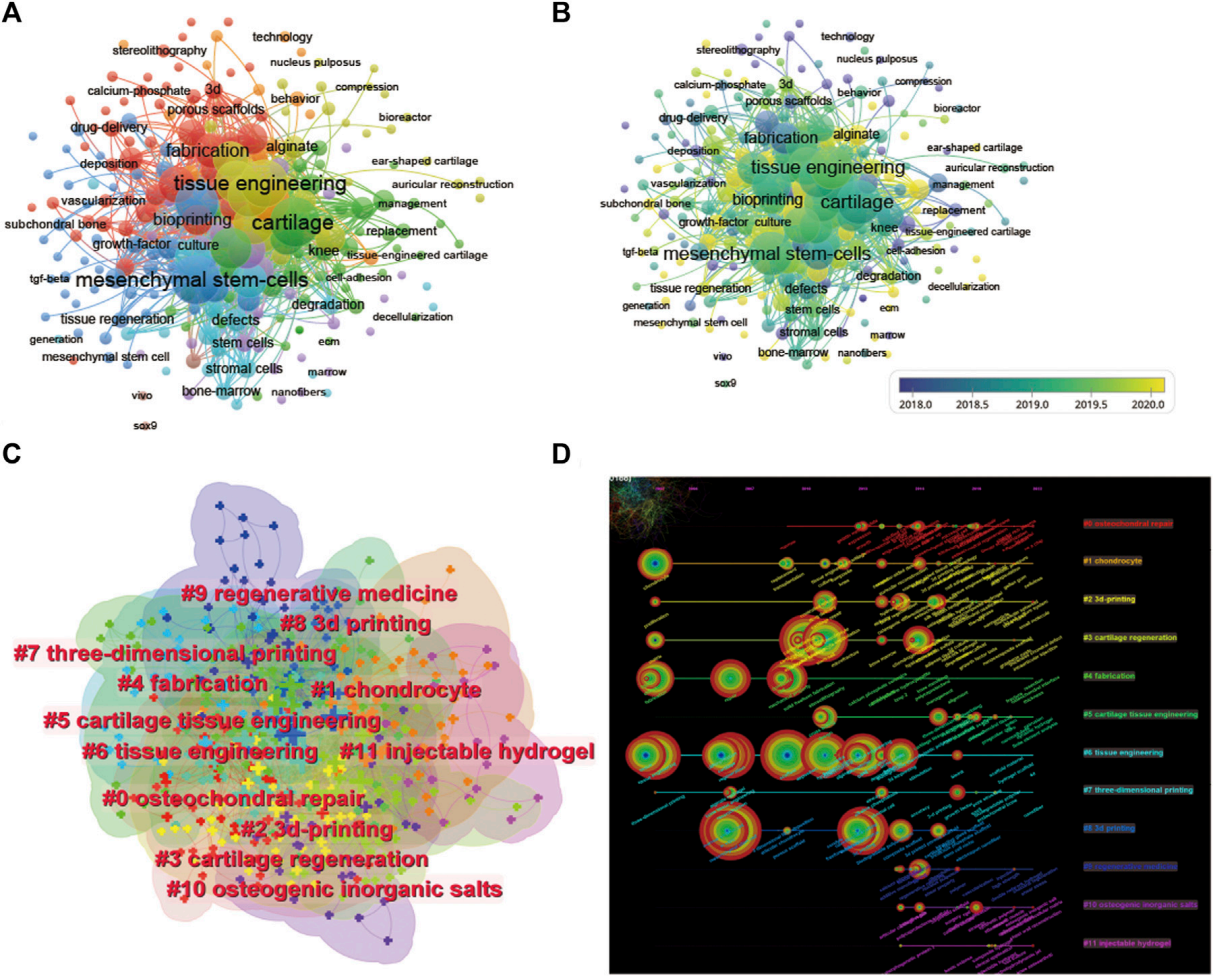
Mapping of keywords in studies on 3D printing in cartilage regeneration and repair. **(A)** Network visualization of keywords by VOSviewer. **(B)** Distribution of keywords according to average publication year (blue: earlier, yellow: later) by VOSviewer. **(C)** Clustering analysis of the keyword network based on CiteSpace. **(D)** Keyword timeline visualization from 2002 to 2022 by CiteSpace.

We performed a network visualization of the keywords of the collected articles (Figure 8A). Among the 2,674 keywords, the top five keywords with the highest total connection strength were mesenchymal stem cells (total connection strength = 1,560 times). Tissue engineering (total link strength = 1,537 times), cartilage (total link strength = 1,536 times), 3D print (total link strength = 1,339 times) and scaffolds (total link strength = 1,216 times). We further visualized the average publication year of the keywords based on this (Figure 8B). We established a visual cluster of keywords through cluster analysis and found that “osteochondral repair” (cluster 0), “chondrocyte” (cluster 1), “3D-print” (cluster 2), “cartilage regeneration” (cluster 3), and “fabrication” (cluster 4) have been research hotspots since 2002 (Figure 8C). We further time‒axialized the above keywords to observe the change in keyword popularity in the temporal dimension (Figure 8D).

## 4 Discussion

### 4.1 Publication trends in the field of 3D printing cartilage regeneration

Our team conducted a bibliometric analysis of papers from 2002–2022 to explore the progress and future direction of the field. The number of publications in the field of 3D printing cartilage regeneration worldwide increased every year between 2002 and 2022. After 2014, there was a sharp increase in the number of papers on 3D printing cartilage regeneration worldwide. This leads us to speculate that this trend will peak approximately 2025, when the field will enter its golden age. Looking at the world, China and United States have far more publications than any other country/region, with the two together accounting for more than 60% of the world’s publications. This is inextricably linked to the strong financial investment in the field by both countries. Eight of the top ten funding agencies are from China and the United States. Interestingly, although China has more publications than United States, United States has the highest H-index and the most citations, suggesting that its articles may have a higher impact. In terms of average citations, however, United States and China are overtaken by other countries in fourth and ninth place, respectively. Chinese officials have taken note of this problem and have proposed measures to improve the quality of academic publications. The top five institutions in the top ten publishers are all from China, and China accounts for six of the top ten authors. This may explain why China has produced a large number of papers on 3D printing cartilage regeneration in recent years. These findings imply that building top-notch research institutions and increasing investment in research are key to improving the country’s academic standing.

Among the top 10 most published journals in this field, the top 5 are Biofabrication, Acta Biomaterialia, Frontiers in Bioengineering and Biotechnology, Adavanced Healthcare Materials, and Biomaterials. Among the top five, Frontiers in Bioengineering and Biotechnology and the sixth to 10th journals had an IF below 10, except for Tissue Engineering Part A and Journal of Biomedical Materials Research Part B Applied Biomaterials, which are all open access journals established in recent years. We speculate that the current trend of authors preferring to use open access channels may be because their publications can reach a wider audience and that these journals may have faster editorial review than “traditional” journals from more established publishers. The top 10 journals consistently have a much higher number of citations per article than IF, suggesting that articles in the field of 3D printing cartilage regeneration have played a positive role in increasing the journal’s IF. One of the most notable journals is Frontiers in Bioengineering and Biotechnology, which has the third highest number of articles and the second highest number of single citations (70.13), despite its IF of 6.064. This indicates that the articles in this journal are more representative and attract the attention of editors and readers, and it is recommended that additional articles be submitted to such publications.

Not surprisingly, the top 10 journals are all related to materials due to the relevance of 3D printing technology to biomaterial design and processing. Of the top 10 representative research areas, eight belong to the broad field of physical and chemical sciences and three to the biological sciences, indicating frequent interdisciplinary interactions. The biplot analysis shows that research is concentrated in materials, medicine and physical chemistry.

The peak in citations of the top 15 cited scholars and the top 15 cited papers in the field both started after 2012, which may be related to the larger innovations in 3D printing technology at that time. Collaboration analysis shows that research relationships between authors in the field tend to be limited to the same country, suggesting the need for more international collaboration in the field. The most cited review in this field was the review Recent advances in 3D printing of biomaterials published in 2015 in the Journal of Biological Engineering, followed by a review on bone tissue engineering scaffolds published in 2017. The top 5 most cited articles typically focused on topics such as cartilage regeneration and preclinical experimental studies of 3D printing scaffolds. These popular topics were validated by a cocitation analysis of the literature on the included studies, grouping the studies into 25 clusters, mainly related to scaffold materials, mechanisms and manufacturing strategies.

### 4.2 Research hotspots and frontiers

The co-occurrence analysis of keywords and emergent phenomena by bibliometrics can identify research hotspots and emerging directions in 3D printing cartilage regeneration, which is crucial for understanding the field. The single most strongly cited keyword can predict where the application of 3D printing in cartilage regeneration may be headed. The co-occurrence network of keywords reflects all keywords incorporated in the titles/abstracts of publications, which we summarize into three main sections: 3D bioprinting, cartilage regeneration, and tissue engineering.

#### 4.2.1 3D bioprinting

3D bioprinting is a method that allows the production of three-dimensional objects through the spatiotemporally controlled deposition of successive layers of biological materials or cells and is capable of producing personalized structures with precisely controlled mechanical properties and physiological heterogeneity. The bioprinting process is usually divided into three steps: 1) design modeling: CT and MRI techniques are used to obtain data on the characteristics of biological tissues or organs, and 3D models are constructed with the help of computer-aided design and computer-aided manufacturing (CAD-CAM); 2) bioink selection: bioinks for tissue or organ repair are prepared; and 3) print reconstruction: bioprinters are used to construct natural tissues or organs in 3D (Matai et al., 2020; Ouyang et al., 2020). Several bioprinting methods have been used for tissue engineering, including inkjet printing, extrusion printing, laser-assisted printing, light-cured printing, and digital light processing (DLP) 3D printing. These methods work on different principles and therefore have different application areas (Wang et al., 2022).

#### 4.2.2 Cartilage regeneration strategy

Cartilage consists of chondrocytes and a large amount of ECM. The main structural components of cartilage ECM are proteoglycans and collagen, distributed in different regions and bands of tissue, forming a network of microfibrils with shock-absorbing properties and resilience to stress. The tensile strength of cartilage is attributed to collagen, the compressive stiffness is attributed to proteoglycans, and cell-matrix interactions are regulated by noncollagenous proteins. The lack of blood vessels and lymph in cartilage tissues, the weak regenerative capacity of cartilage, and the damage once it occurs are mostly incurable (Bhosale and Richardson, 2008; Sophia Fox et al., 2009). Therefore, there is a growing need to construct regenerative cartilage with mechanical and ontogenetic characteristics similar to those of natural cartilage (Christensen et al., 2015). 3D printing of cartilage using multiple types of biomaterials and cells and patterning them into defined structures has shown translational potential for repairing cartilage with clinically relevant dimensions and geometry. To mimic the ECM composition of natural articular hyaline cartilage, several biomaterials, including natural macromolecules, synthetic polymers, and hybrid biomaterials, have been applied for 3D printing of cartilage (Shi et al., 2016). Among them, gelatin methacrylated (GelMA), a gelatin derivative that combines the biocompatibility of natural ECM with the stability, reproducibility, and modularity of synthetic biomaterials, has been widely used for 3D printing of articular hyaline cartilage (Daly et al., 2017; Hong et al., 2020). In addition to GelMA, cartilage matrix components include hyaluronic acid and chondroitin sulfate, which can be integrated with gelatin to form a bionic scaffold capable of supporting superior new cartilage formation (Yue et al., 2015).

#### 4.2.3 Tissue engineering

Tissue engineering aims to generate functional substitutes to restore or replace tissues damaged by injury or disease. However, regenerating three-dimensional tissue structures with clinically relevant size, shape, and structural integrity remains a major research and clinical challenge due to the complex tissue architecture of cell types, extracellular matrix (ECM) components, and biologically active agents in natural tissues (O'Shea et al., 2022).

### 4.3 Prospects for 3D printing cartilage regeneration

In the last 20 years, the field of 3D printing cartilage regeneration has developed rapidly in terms of production technology, material selection, and tissue regeneration strategies. However, there are still some issues that need to be addressed on the road to clinical translation in the future: 1) The heterogeneity of cartilage layers, including the morphology and arrangement of cells and the composition and distribution of extracellular matrix, requires the application of more advanced 3D printing technologies to construct precise and complex cartilage regeneration scaffolds from a microscopic perspective. The inability of current single 3D printing materials to simultaneously meet multiple requirements, including good cytocompatibility, controlled biodegradability, good mechanical properties, and excellent chondrogenic bone differentiation, has hindered their translation to clinical applications. The combination of multiple printing materials may provide a completely new way of thinking (Zhao et al., 2015). 2) Cartilage regeneration strategies should also be more innovative, and the cells and factors incorporated on the basis of 3D printed scaffolds can be more developed. Currently, the most common seed cells have inconveniences, such as invasive material extraction and difficult access. Therefore, the selection of seed cells can be transferred to urine-derived stem cells, iPSCs and other cells. There are also many innovations in factor selection, such as the sequential release of multiple factors, on-demand release, and intelligent responsive release. 3) The molecular and cellular mechanisms of cartilage regeneration are still not well studied, and the specific network of action of each regeneration stage is not clear (Gasperini et al., 2015). 3D printing scaffolds that conform to each stage of cartilage regeneration is a future goal. Current 3D-printed cartilage regeneration scaffolds focus on immunomodulation, particularly the inhibition of proinflammatory M1 macrophages and promotion of anti-inflammatory M2 macrophages. However, there is an active role of M1 macrophages in repair, while excessive infiltration of M2 macrophages may be detrimental to cartilage regeneration. Therefore, an in-depth exploration of cartilage regeneration mechanisms will provide a novel pathway in the field of 3D printing cartilage regeneration.

### 4.4 Strengths and limitations of the study

This study used bibliometric and visual analysis to explore the literature on the field of 3D printing cartilage regeneration over the last 20 years. Our findings are relatively comprehensive and objective, but there are some unavoidable limitations. First, all publications in this study were extracted from the Web of Science Core Collection (WOSCC), and no literature searches were conducted using the PubMed, Scopus, Cochrane, and Embase library databases. Although WOSCC is considered to be one of the most commonly used and authoritative comprehensive databases, it is still possible that some literature relevant to this study was not included, leading to selection bias. Second, this study excluded non-English language articles and nonresearch/review articles, thus missing a large number of relevant studies published in other languages, with a significant contribution from Chinese publications in this area. Reviews and research articles are both useful publication types, both important and valuable, serving their unique purposes. Therefore, we do not discuss research articles and reviews separately here. In addition, articles published after December 2022 are not included, which may lead to a certain degree of prediction bias when conducting the correlation analysis. Finally, we failed to include the quality of publications as a factor in some analyses, giving equal weight to high-quality and low-quality publications.

## 5 Conclusion

This study provides the first comprehensive bibliometric and visual analysis of 3D printing research in the field of cartilage repair and regeneration, showing the research dynamics in this field over the past 20 years. We systematically analyzed global trends in this field of research and identified influential authors, institutions, and journals. In addition, through the co-occurrence analysis of key words and emergent phenomena, we can identify the research hotspots and emerging directions mainly in the three categories of “3D bioprinting,” “cartilage regeneration strategy” and “tissue engineering technology” and creatively summarize these three directions. This study systematically and comprehensively summarizes the research status of 3D printing in the field of cartilage repair and regeneration, summarizes the research hotspots, and predicts the development trend. It will help to shape researchers’ understanding of 3D printing and cartilage repair and regeneration, inspire researchers’ research ideas, guide research directions, and promote related research results to clinical application.

## Impact statement

This review article systematically and comprehensively describes the current research status of 3D printing in cartilage repair and regeneration, and predicts the development trend of 3D printing, which can help researchers to deepen their understanding and raise awareness, promote the development of this research field, and push related research towards clinical application.

## Data Availability

The raw data supporting the conclusion of this article will be made available by the authors, without undue reservation.
